# A National Cyberattack Affecting Radiation Therapy: The Irish Experience

**DOI:** 10.1016/j.adro.2022.100914

**Published:** 2022-08-06

**Authors:** Aileen Flavin, Eve O'Toole, Louise Murphy, Ruth Ryan, Brendan McClean, Clare Faul, Carol McGibney, Stephen Coyne, Geraldine O'Boyle, Cormac Small, Caroline Sims, Maeve Kearney, Mary Coffey, Anita O'Donovan

**Affiliations:** a Department of Radiotherapy, Cork University Hospital, Cork, Ireland; b National Cancer Control Programme (NCCP), King's Inns House, Dublin, Ireland; c St. Luke's Radiation Oncology Network (SLRON), Dublin, Ireland; d Department of Radiotherapy, University Hospital Galway, Galway, Ireland; e Applied Radiation Therapy Trinity (ARTT),Trinity College Dublin, Trinity St. James's Cancer Institute, Dublin, Ireland; f ESTRO Radiation Oncology Quality and Safety Committee (ROSQC), Brussels, Belgium

## Abstract

On Friday, May 14, 2021, the Health Service Executive, the organization providing public health services in the Republic of Ireland, was the victim of a significant cyberattack on its information technology systems. All systems were subsequently shut down to prevent further damage and to allow cybersecurity experts to investigate the attack. As a result, oncology services were severely disrupted, with the cessation of radiation therapy treatments in all public radiation therapy departments. Ireland has 5 large public and 6 smaller private radiation therapy centers in total. Because of the widespread adoption of electronic medical records in radiation therapy departments, it wasn't possible to retrieve patient details of those who were undergoing radiation therapy at the time of the cyberattack. In total, 513 patients nationally had their radiation therapy interrupted.

A national radiation therapy cyberattack response team was formed immediately to oversee the response to the attack. The immediate concerns were radiation therapy emergencies and category 1 patients where gaps in treatment would have an adverse effect on outcome. Communication with patients and the public was also established as a priority and agreements were reached with the private sector for the treatment of patients affected by the cyberattack. The national media was used to alert patients of the need to communicate with their radiation therapy department. Dedicated phone lines were established. Locally, radiation therapy departments held daily crisis meetings with key staff members, including information technology personnel. Individual centers employed different technologies for treatment planning and data storage, so local solutions to the cyberattack to reestablish radiation therapy for patients were developed. In addition, national documentation on prioritization of patients to resume treatment was produced and a national approach was made to compensate for gaps in treatment caused by the attack. All 5 centers had reestablished radiation therapy by May 30, although there has been a long aftermath to the cyberattack. In this article, we provide an overview of the effects of the cyberattack on our national radiation therapy service and our strategy to resume patient treatment in a timely fashion.

## Introduction

The Health Service Executive (HSE) in the Republic of Ireland is responsible for the delivery of public health and social care to the population of the Republic of Ireland, which serves approximately 5 million people. The Irish National Cancer Control Programme (NCCP) was established in 2007 as a directorate within the HSE. The objective of the NCCP is to implement the National Cancer Strategy and oversee cancer services.^1^ There are currently 5 hospitals providing public radiation oncology services in the Republic of Ireland: Dublin St. Luke's Radiation Oncology Network (SLRON), which includes (1) St. Luke's Hospital, (2) SLRON at St. James's Centre, and (3) SLRON at Beaumont Centre; (4) Cork (Cork University Hospital (CUH)); and (5) Galway (Galway University Hospital [GUH]). There is also a significant private sector in Ireland, which was of major importance during and in the aftermath of the cyberattack.

Radiation oncology practice is continuously evolving with sophisticated imaging and planning and delivery system technologies. Management and storage of large volumes of planning and image data sets has led to paperless environments and the use of electronic medical records (EMRs) becoming commonplace in radiation therapy departments. This reliance on technology means radiation therapy services are particularly vulnerable to cyberattacks, which have been increasing worldwide,^2^ with cyberattacks disrupting radiation therapy services in the United States and New Zealand most recently.^3^, ^4^, ^5^, ^6^

On Friday, May 14, 2021, at 1 AM it was discovered that the HSE was the victim of a significant cyberattack on its information technology (IT) systems, via Conti Ransomware.^7^^,^^8^ As a result, over 80% of IT infrastructure was affected in the public health service as a whole, with the widespread loss of patient information and diagnostics. This resulted in severe effects on the national health service and the provision of care, including oncology. National communication systems were also lost, including telephone networks. The HSE immediately invoked its critical incident process and secured the assistance of the National Cyber Crime Bureau police service, the International Criminal Police Organisation (Interpol), and the National Cyber Security Centre. On the day of the cyberattack, 31 of the 54 acute hospitals within the HSE announced cancellations of at least some of their services.^8^ The source of the cyberattack originated from a malicious software “malware” infection on a HSE workstation. This occurred as a result of the user of the workstation opening an Excel file that was attached to a phishing email sent to the user on March 16, 2021.^8^ The effect of this attack caused widespread disruption of services, which varied by hospital and included outpatient clinics, diagnostic imaging, radiation therapy, laboratory investigations, and elective surgeries.

In terms of cancer services, medical and surgical oncology were not as severely affected as radiation therapy, as they were not entirely reliant on EMRs. As electronic prescribing of chemotherapy had not yet been established nationally, it meant there was little disruption to chemotherapy delivery, apart from concurrent chemotherapy being stopped until patients resumed radiation therapy. Time-dependent cancer surgery went ahead as planned. The reestablishment of radiation therapy and radiology services became a national priority for the HSE during the cyberattack due to the effect on clinical service delivery. This paper outlines our account of the cyberattack and its aftermath for radiation therapy to assist other departments that may find themselves in similar circumstances in the future.

## Immediate Effect and Initial Management of the Cyberattack

**Fig. 1:**
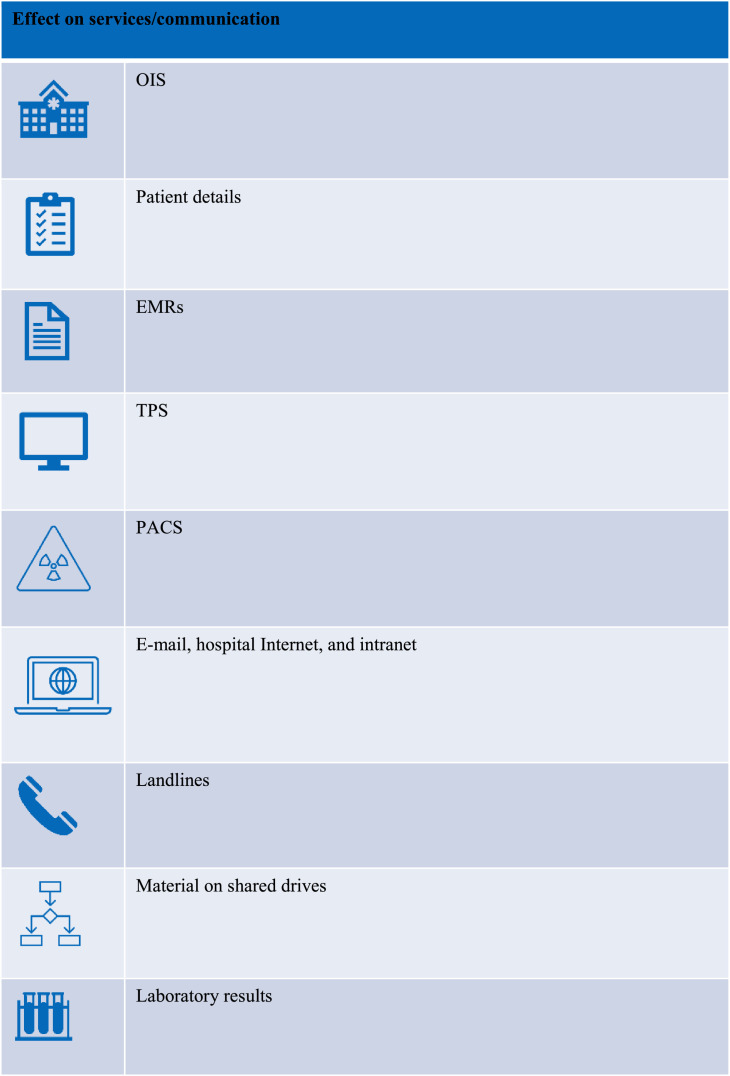
Systems that were shut down due to the cyberattack. *Abbreviations:* EMRs = electronic medical records; OIS = oncology information system; PACS = picture archiving and communications systems; TPS = treatment planning system.

**Fig. 2:**
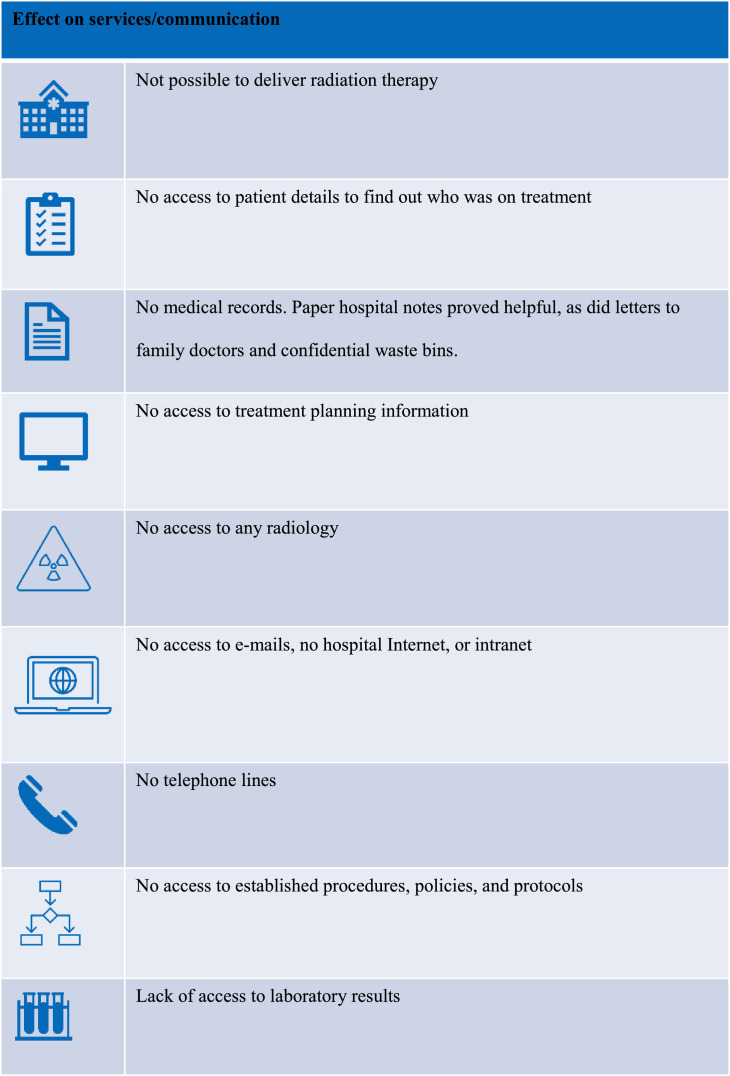
Implications of lost services.

The immediate effect of the cyberattack (Fig. 1, Fig. 2) on the HSE public radiation therapy departments was that all systems were immediately shut down to prevent further encryption of systems and to allow cyber security experts to investigate the attack. Because of the widespread adoption of EMRs in radiation therapy departments, it wasn't possible to retrieve any details of patients who were undergoing radiation therapy at the time of the cyberattack. With no access to IT systems or oncology information systems (OIS), all health care services were severely disrupted, with the cessation of radiation treatments in all 5 public radiation therapy departments for varying lengths of time. It wasn't possible to ascertain which patients were on treatment or how many fractions patients had completed. All information within the OIS and treatment planning systems, including clinical notes, patient demographics, contact details, and radiation therapy planning information, were inaccessible. Radiation therapy delivery was impossible, as an OIS is necessary to download plans to the LINAC. In total, 513 patients nationally had their radiation therapy treatment interrupted. Private radiation therapy facilities were unaffected.

An NCCP radiation oncology team comprising NCCP staff, the lead radiation oncologist for each center, the national radiation oncology clinical advisor, national medical physics lead, radiation therapist, and nursing leads, all met daily. Representatives from the national HSE IT staff also attended these meetings. This team, which had met regularly since the COVID-19 pandemic began, became the Radiotherapy cyberattack response team and met daily until radiation therapy services were reestablished in all centers.

The top priority for this team was to provide treatment for radiation therapy emergencies and to reestablish radiation therapy for patients classified as category 1, for example, patients with head and neck or cervical squamous cell carcinomas, where prolonging overall treatment time is known to have an adverse effect on outcome.^9^^,^^10^ These categories were defined according to Royal College of Radiologists criteria.^11^ An agreement was immediately reached with the private sector to treat any patients requiring emergency radiation therapy. In addition, patients who required time- dependent brachytherapy for cervical cancer would be treated at the Mater Private Hospital in Dublin. The NCCP Radiotherapy cyberattack response team held daily meetings focused on how to prioritize patients for treatment in a limited capacity situation, how to compensate for gaps in treatment, and how to reduce the risk for patients and staff when working in suboptimal conditions.

Communication with patients and the public was also established as a priority. The national media and HSE website were used to alert patients of the need to communicate with their radiation therapy department using newly established dedicated phone lines. As patients got in touch the centers were able to establish lists of patients on treatment. Regular communication directly with patients and via the HSE website was used to provide updates about a rapidly evolving situation. An agreement called *Safety Net 2* had been put in place in January 2021 to allow HSE hospitals to use private sector facilities in the case of inadequate capacity during COVID-19 surges. This agreement was modified to include provision of non–COVID-19 capacity during the cyberattack, which facilitated patients to have all treatments including radiation therapy in private centers. The national State Claims Agency clarified they would continue to provide indemnity to HSE staff during the cyberattack.

**Fig. 3:**
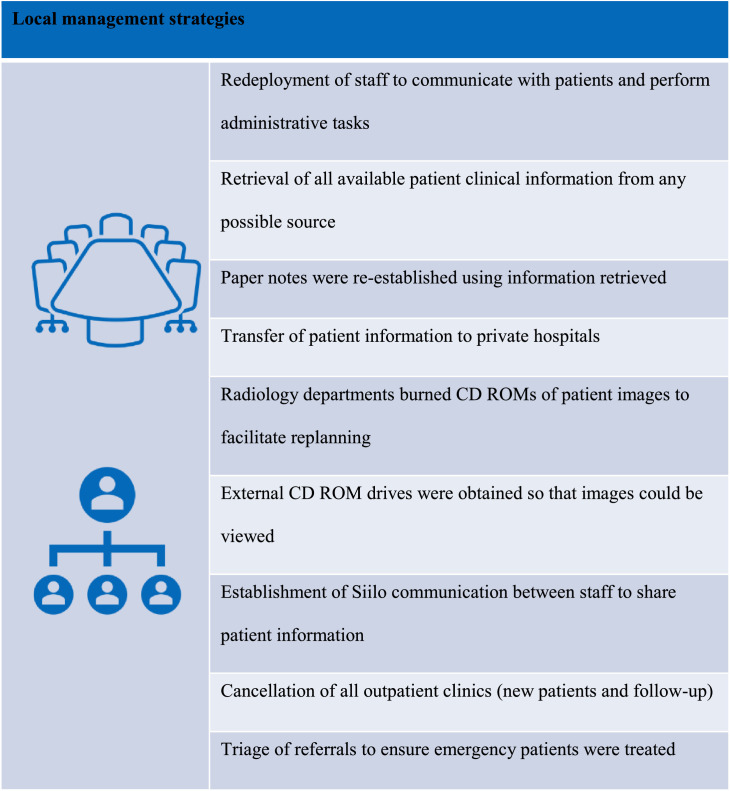
Local management strategies to mitigate against the adverse effects of the cyberattack.

Locally, radiation therapy departments held daily multidisciplinary management crisis meetings with key staff members, including IT personnel. This led to the rapid establishment of incident rooms, which were set up in each department. Staff were redeployed to man these areas. Other local measures taken to mitigate against the adverse effects of the cyberattack included the reestablishment of paper records, requesting pathology reports, liaising with radiology departments to obtain CD ROMs of patient images to facilitate planning, and arranging appointments (Fig. 3). The incident rooms were led by radiation therapists, supported by administrative staff, and involved collating clinical information from patients who called the helplines as well as information from family doctors and other medical professionals. The priority was to retrieve as much information as possible about patients and to triage patients for treatment on a priority basis. This retrieval of patient clinical information was a significant task. Patients were asked how many fractions they had received. Family doctors were contacted for any information they had received from the hospital on individual patient diagnosis and treatment, notes from multidisciplinary patient meetings were retrieved, and confidential waste containers were opened and contents examined, in the hope of finding any information regarding diagnosis and overall management for patients on treatment. Siilo*,* a secure medical messaging app for medical professionals, which is General Data Protection Regulation–compliant, assisted with communicating patient information securely. Obtaining this information was essential for patients who were being transferred to private radiation therapy facilities, as they all required rescanning, redelineation of target volumes/organs at risk, and replanning. During this period, new referrals were triaged to ensure patients requiring emergency radiation therapy accessed care in a timely manner. All radiation oncology outpatient appointments (new patients and follow-up) were cancelled so that staff could focus on resuming treatment for existing patients.

## The Effect of the Cyberattack on Radiation Oncology Centers

Due to different OIS, treatment planning systems, LINACS, and IT arrangements both between and within centers, local solutions to the cyberattack differed (Details in Suplemetary Material Table E1). The media assisted with provision of national helplines so that patients on treatment could contact their radiation therapy departments, as there was no way of retrieving patient contact details. Brachytherapy was less affected than external beam treatments and was reestablished within 10 working days in all centers. Stand-alone orthovoltage units were unaffected.

**Fig. 4:**
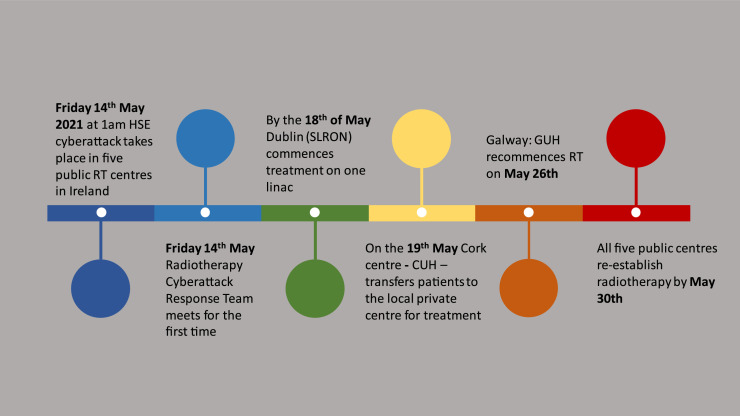
Timeline of events during the national cyberattack.

In the case of the SLRON, all category 1 patients resumed their treatment by May 19, with gaps ranging from 1 to 3 working days from the onset of the cyberattack. Category 2 and pediatric patients had resumed treatment by May 24 with gaps ranging from 3 to 6 working days. For Cork University Hospital, the median gap duration was 6 days (range, 6-17 working days) for category 1 patients, and for category 2 patients the median gap duration was 10 days (range, 6-13 working days). For Galway University Hospital the median gap duration was 6 days (range, 4-9 working days) for category 1 patients and 8 days (range, 7-8 working days) for category 2 patients. All 5 centers had reestablished radiation therapy services by May 30, 11 working days after the onset of the cyberattack (Fig. 4). New patients were being scanned for planning radiation therapy by June 8, 2021 Centers were then at 50% capacity, treating some adjuvant patients and new referrals for radiation therapy. The need for support from private centers continued, however.

## Prioritization of Patients to Resume Treatment

**Fig. 5:**
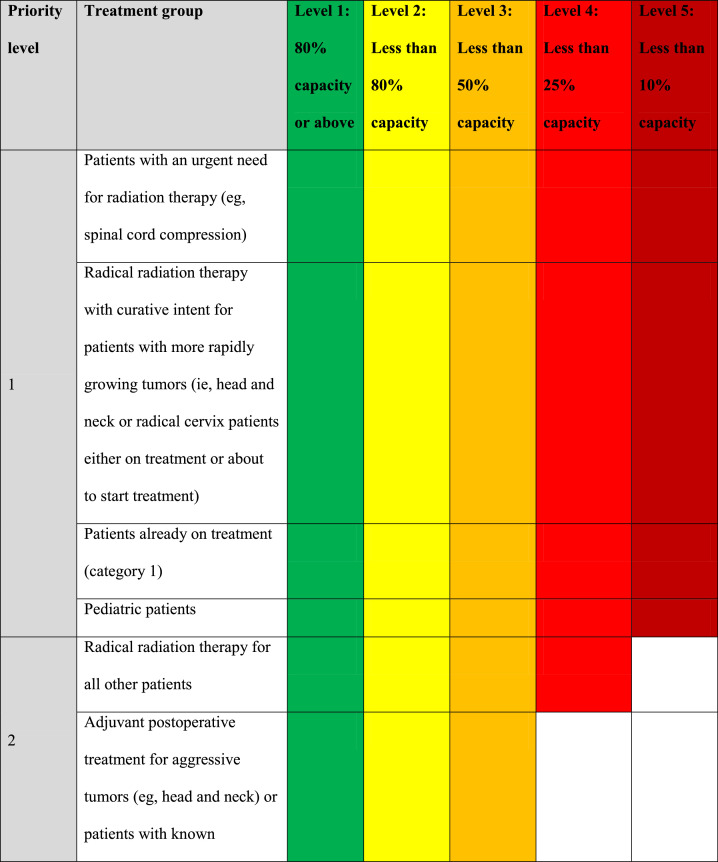

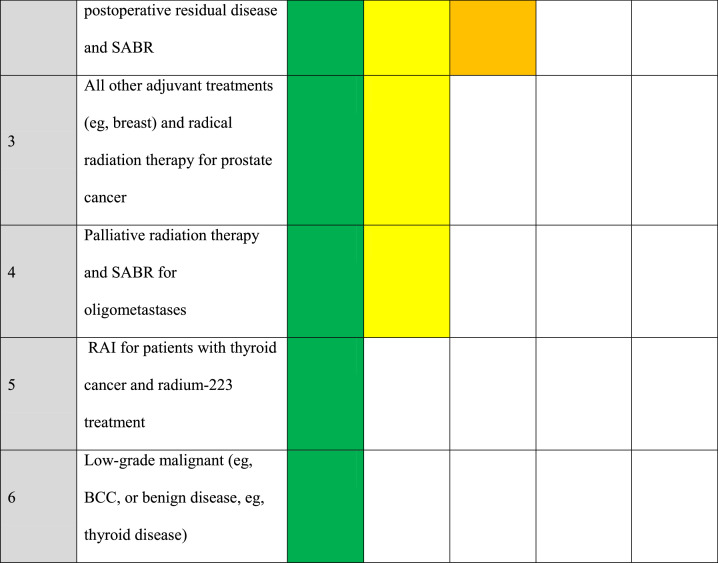
The National Cancer Control Programme's capacity escalation plan as its radiation oncology response to the cyberattack. *Abbreviations:* BCC = basal cell carcinoma; RAI = radioactive iodine.

The NCCP document on prioritization of patients to resume treatment (Fig. 5) was produced early so that decisions could be made easily and would be nationally consistent. This was produced by the multidisciplinary NCCP Radiotherapy cyberattack response team, by consensus. Consistent with the preexisting COVID-19 capacity document,^12^ category 1 patients, pediatric cases, and those patients who had already started treatment were prioritized for treatment.

## Compensation for Gaps in Treatment

The NCCP document regarding compensation for gaps in treatment during COVID-19 was adopted for the cyberattack.^13^ The physics team from GUH shared their specially designed MS Excel sheet for calculating the additional dose to be delivered. Based on these calculations, patients were treated 6 days a week once radiation therapy recommenced, therefore additional compensation doses tended to be low. For many patients, the calculated extra doses required were of the order of 2 Gy, and adding another fraction of radiation therapy was a common strategy. Compensation was more difficult for patients nearing the end of treatment and where the additional suggested doses were high, and therefore clinical judgment had to be applied. No correction was made for palliative patients having shorter courses of treatment or those receiving hypofractionated breast/chest wall radiation therapy receiving the FAST-forward regimen (26 Gy/5).^14^

## Risk Assessment of Actions Taken During the Cyberattack

**Fig. 6:**
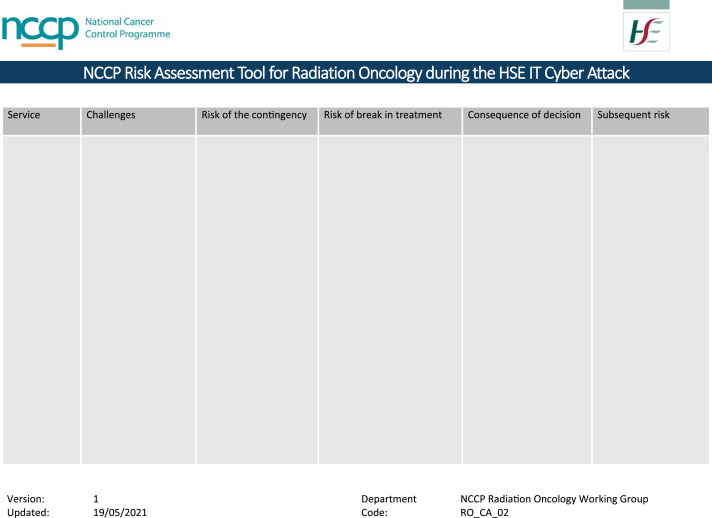
National Cancer Control Programme (NCCP) risk assessment tool for radiation oncology during the Health Service Executive (HSE) information technology (IT) cyberattack.

The NCCP Radiotherapy cyberattack response team felt it was important to create a risk assessment template for all patients having their treatment replanned in another center (Fig. 6). Examples of completed templates are provided in the Supplementary Materials for a patient with lymphoma (Fig. E2a), a patient treated with palliative intent (Fig. E2b), a patient with cervical cancer (Fig. E2c), and a patient with node-positive breast cancer (Fig. E2d). The risk of replanning patients without all the essential information normally available had to be weighed against the risk of exposing the patient to the consequences of large gaps in their treatment. Lack of access to imaging meant the utilization of less complex forms of treatment planning, such as 2- or 3-dimensional conformal radiation therapy rather than intensity modulated radiation therapy/volumetric modulated arc therapy, needed to be considered. In this scenario the team felt it was important to document the reasons for using simpler forms of radiation therapy that could increase the risk of a treatment-related late complication.

**Fig. 7:**
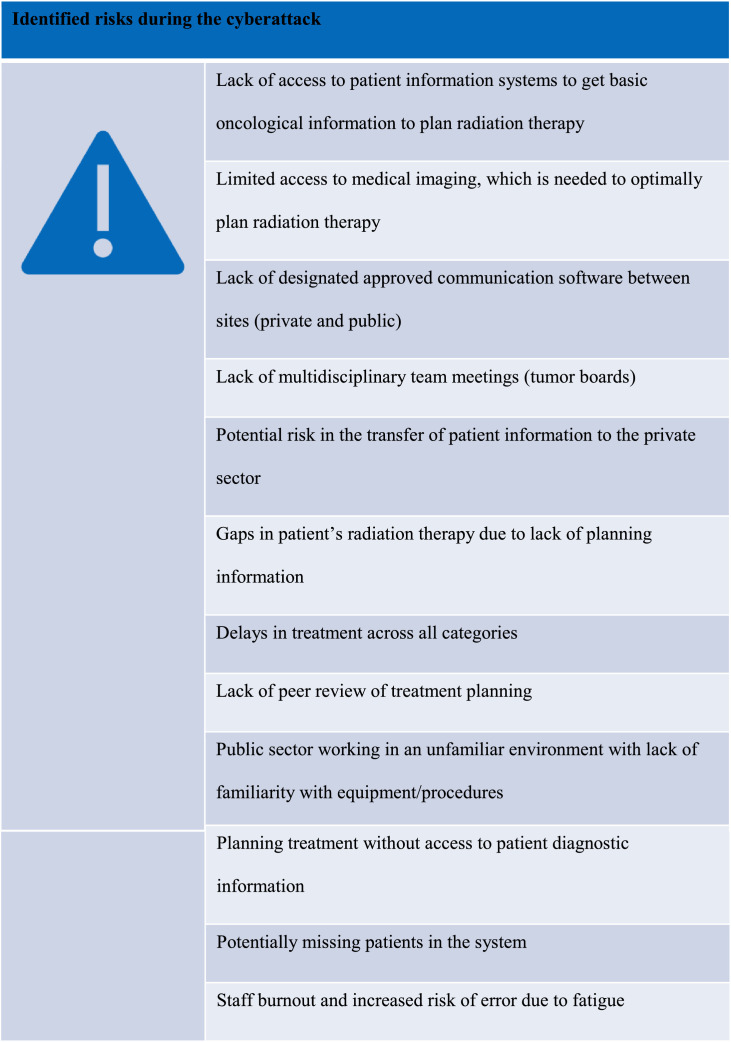
National Cancer Control Programme–identified risks during radiation therapy cyberattack.

The NCCP group identified some of the many risks experienced during the cyberattack, such as lack of access to patient information and imaging systems, lack of communication infrastructure, risks associated with patient transfers to the private system, delays and gaps in treatment, and working in unfamiliar conditions with limited information to assist with treatment planning (Fig. 7).

## The Aftermath of the Cyberattack

The first 2 weeks of the cyberattack were the most difficult period for patients and staff. The workload in replanning patients who needed to resume treatment in another center was enormous. Staff manning incident rooms over a number of days succeeded in retrieving information necessary to replan patients. This resulted in no patients having less complex planning techniques than originally intended. The situation gradually improved as all existing patients resumed treatment, and it also became possible to start new patients on treatment. It was, however, far from perfect, with systems operating slowly. Reestablishing e-mail communication took several weeks, it took 4 months to gain consistent access to the picture archiving and communications imaging systems (which meant continued reliance on burning CD ROMs for assessing patients and planning radiation therapy), and it was 5 months after the attack before remote access was restored. The private hospitals continued to provide additional patient treatment services to the HSE.

## Discussion

The well-known aphorism used during the COVID-19 pandemic, “The perfect is the enemy of good” (Voltaire), holds true for a cyberattack in radiation therapy, where fast action is needed to get patients back on treatment in a far from perfect situation. The occurrence of a national cyberattack affecting radiation therapy was devastating, with all patient information and treatment capacity unavailable. This cyberattack in Ireland is, to our knowledge, the only example of one that affected a national public health care service.^8^ It was helpful for radiation therapy that the NCCP radiation oncology team was already established, accustomed to working as a team, and able to quickly adapt to the new crisis. Some of the tactics used in Puerto Rico during Hurricane Maria, namely PCOC (prepare, communicate, operate, and compensate) were used.^15^ The difference between a cyberattack and many natural disasters, however, is that there is no warning, so advance planning for the event or ideally making your departments more resilient to a cyberattack is required.

The experience of the recent cyberattacks in the United States, putting enormous pressure on individual radiation therapy departments and the consequent staff fatigue, closely mirrored the Irish situation.^4^^,^^5^ There was no normal family life for radiation therapy staff during the first 2 weeks of the cyberattack due to long working hours. Similarly, it took several days to resume radiation therapy, and full recovery with access to essential systems took months. Likewise, Harrison et al^6^ recount the experience in the United States of a ransomware attack against a major radiation oncology record and verify system, which affected multiple centers across the country in April 2021. This resulted in disruption of the radiation oncology service, albeit for a relatively short duration, as the attack was limited to the record and verify system and they implemented a backup plan quickly. Their experience also highlights the duration and aftermath of an attack, as it took 4.5 weeks for them to get back to full functionality.^6^

As with many critical situations, the response by both staff and patients was admirable. Working long hours and attending treatment at unsocial hours are examples of the resilience of staff and patients. Efforts by the private sector enabled treatment to quickly restart for emergency and category 1 patients. A full HSE report on the cyberattack and the lessons to be learned for the health service as a whole have been published, which summarizes their findings under the themes *prepare, response*, and *recovery*.^8^ This report highlights the need for investment in cybersecurity to maintain a secure, resilient modern IT structure in the future. We have adapted this thematic approach to summarizing the specific lessons learned for radiation therapy (Table 1).

**Table 1:** Summary of lessons to be learned for radiation therapy

Theme	Lessons to be learned
Prepare	Governance for cybersecurity must be established to ensure the risks associated with radiation therapy are actively managed, including the resilience of the department to a potential cyberattack.
	Departments should put in place a cyber security strategy, identifying the potential risks and how they may be mitigated.
	Individual departments should regularly risk assess their respective areas in line with an effective cyber security strategy, under the guidance of a suitably appointed expert in the field.
	Radiobiological guidelines and formulae are needed for such unexpected and prolonged gaps in radiation therapy treatment and how they may be adequately compensated for in each cancer site. Expert groups should be set up to address this in the event of future cyberattacks or other unforeseen interruptions in radiation therapy delivery.
	Contingency planning should be in place by having some back-up information on a separate computer, so that details of patients currently on treatment, protocols, policies, procedures, etc, can be accessed.
	Being able to store one's own radiation therapy data, rather than relying on hospital servers, as well as having an in-house team with knowledge and understanding of IT related to radiation therapy, is a necessity in radiation therapy departments.
	Build relationships with other radiation therapy providers (nationally, locally, and in the private sector). Develop contingency plans between departments. Such relationships are vital in the event of a cyberattack or other disruptions to service, to facilitate resumption of patient treatments and to deal with aftermath when capacity is still low.
	Radiation therapy departments should develop cybersecurity-specific crisis management plans detailing the actions required in the event of such an attack.
	Regular staff education programs should be conducted on key methods to protect against cyberattacks, for example, from phishing, as was the case for the HSE incident.
	Store a list of patients currently scheduled for treatment separate from departmental IT systems so that no time is lost in accessing basic patient information in the event of a cyberattack.
Response	Set up local and national multidisciplinary teams, including cyber security personnel, to lead the management of the cyberattack.
	Set up a dedicated incident room, involving the multidisciplinary team.
	Collaborate with national media to facilitate early and ongoing communication with patients.
	Collaborate with vendors regarding possible solutions, for example, remote planning assistance.
	Set up a helpline or other communication channel for patients to communicate with the radiation therapy department.
	Use existing patient prioritization frameworks to select patients who urgently require re-establishment of treatment as soon as possible.
	Incorporate simpler planning methods, where appropriate, to expedite the process of resumption of treatment.
Recovery	Assess IT security on an ongoing basis to remain up to date with current protective measures.
	Invest in a cyber security team and resources for radiation therapy.
	Ensure patients are empowered to hold data in relation to their radiation therapy treatment progress, for example, number of overall treatments planned and number delivered to date.
	Communicate with patients who have been affected to ensure they know that measures are being put in place to prevent such an attack in the future.

*Abbreviations:* HSE = Health Service Executive; IT = information technology.

We need to acknowledge that we do not know either the oncological or psychological effect that the cyberattack had/will have on patients whose radiation therapy course was interrupted. Our radiobiological formulae for dealing with gaps in radiation therapy treatment were never designed for the long gaps experienced by some of our category 1 patients. Even for shorter gaps in treatment we are reminded that radiobiological calculations to compensate, which are based on linear quadratic models, are best fit models, and local control and normal tissue morbidity may be adversely affected when using them.^11^ However, some of the site-specific recommendations for compensating 2- to 3-week gaps in radiation therapy from Gay et al,^15^ based on their experience of Hurricane Maria in the United States in 2017, are helpful in this context. Replanning radiation therapy in another center poses risks to patients, particularly in the absence of information, as does being treated by staff who are working long hours and/or with unfamiliar equipment. There is a role for vendors in the recovery after a cyberattack; however, vendors were unable to provide immediate support as remote access to systems was disabled. COVID-19 travel restrictions also caused delays to their staff (all based outside Ireland) reaching the centers. An example of vendor support is provided in the Supplementary Materials in the experience of 1 particular center.

We need, as a radiation oncology community, to accept that cyberattacks are now a fact of life. Contingency planning in the short term, by having some backup information on a separate computer so we at least have details of patients currently on treatment, protocols, policies, procedures, and so on, is important. Patients having a copy of their treatment information and planning staff having Excel lists with some basic information on individual patient plans are recommended in preparation for a cyberattack.^4^^,^^15^ Access to key dosimetric data off line would also be helpful. What is particularly needed in radiation therapy departments is the development of robust IT pathways with dedicated secure servers, rather than reliance on hospital systems.^16^ Investment in cybersecurity is of course crucial to ensure that cyberattacks are prevented.

It is our opinion that being able to store your own radiation therapy data, rather than relying on hospital servers, as well as having an in-house team with knowledge and understanding of IT related to radiation therapy are necessities going forward in radiation therapy departments. All of these strategies will come at a cost, but overall, we feel that is a price worth paying given the devastating effect of cyberattacks on radiation therapy services.

## Appendix Supplementary materials

Image, application 1
