# Supplementary material for: A National Cyberattack Affecting Radiation Therapy: The Irish Experience
Source: Adv Radiat Oncol. 2022 Aug 6;7(5):100914. doi: 10.1016/j.adro.2022.100914 (PMC9486432; doi:10.1016/j.adro.2022.100914)
Supplement: Supplementary file 1 [file mmc1.docx]

Individual Centre Response to the Cyberattack

Dublin - St Luke’s Radiation Oncology Network (SLRON): There are 14 linacs in this network. SLRON at St James Centre (SJC) and SLRON at Beaumont Centre (BC) each have 4 linacs respectively with Aria OIS and Eclipse Treatment Planning Systems (TPS). St Lukes Hospital (SLH) has 2 Varian and 4 Elekta linacs with Aria, Mosaic OIS, OMP and Monaco TPS. At the time of the cyberattack there were 304 patients on treatment in the SLRON and 17 due to start. During the first weekend, they successfully isolated Aria to a new, clean computer at the SJC site. This gave staff access to all the patients details and they were able to identify all of the high priority category 1 patients and paediatric cases on treatment. Treatment plans for BC and SJC patients were retrieved. By the 18^th^ of May (3^rd^ working day after attack) they had commenced treatment on one linac and managed to resume treatment for all Category 1 patients by the 19^th^ of May. Paediatric patients who are only treated at the SLH site, were replanned for treatment at the SJC site, and by 24^th^ May all paediatric patients had recommenced radiotherapy. Once SLRON had one linac in operation they were able to re-establish radiotherapy treatments in the remaining linacs at SJC and BC. Gradually in the next 2 weeks patients commenced treatment as per the agreed prioritisation of patients. Nationally, in the public radiotherapy service, SABR and SRS treatments are only delivered in SLRON and initially SABR and SRS treatments were stopped. SABR was re-established within 2 weeks, however SRS was slower to get up and running. Overall the SLRON network was quickest to re-establish radiotherapy and referred fewer patients to the private sector. Unlike the other radiotherapy centres nationally, the SLRON are unique in hosting their own IT data. They also have dedicated IT staff for radiotherapy in their network.

Galway University Hospital (GUH): This department has 3 Siemens linacs with Mosaic and Monaco OIS and TPS. At the time of the cyberattack there were 73 patients on treatment and 27 due to start treatment. GUH had more patient information than other sites as they kept a list of patients on treatment on a USB and admin staff maintain some paper records of patient details. A big issue for GUH was that the local private centre, the Galway Clinic only has one linac, therefore they only had capacity to treat an additional 20 patients. This created a major issue in getting high priority patients back on treatment. On the 21^st^ of May, when there was no indication of when radiotherapy would be re-established in GUH, the Radiotherapy Cyberattack Response Team decided the best course of action was to transfer all remaining Category 1 patients from GUH to the SLRON and replan their treatment during the week commencing 24^th^ May. Fortunately, progress was made with IT that weekend, and a transfer of patients to Dublin was not required as GUH recommenced patients’ treatment on May 26^th^. GUH had a further challenge to service provision caused by a flood in one of the linac rooms on 7^th^ June 2021, which reduced them to 10% capacity temporarily.

Cork University Hospital (CUH): This department has 5 Elekta linacs, with Mosaic and Monaco OIS and planning systems. At the time of the cyberattack there were 136 patients having external beam radiotherapy and 25 patients who had been planned to commence treatment. Agreement was reached on May 19^th^ with the local private hospital, the Bon Secours/UPMC Hillman Cancer Centre (a two-linac centre) to allow one linac to be used by the HSE, and this gave capacity to treat 70 patients per day. An extended working day was required and staff from CUH were redeployed to this centre. Knowing assistance would be required with replanning large numbers of patients, the Bon Secours/UPMC Hillman centre considered remote dosimetry/medical physics services and contacted Varian CTSI (Cancer Treatment Services International) Oncology Solutions. Within 36 hours, CTSI had: i. assembled a team of dosimetrists globally to provide remote planning services ii. arranged the approval of temporary Eclipse licences to facilitate collaboration between radiotherapy staff in Cork and CTSI personnel, and iii Varian’s legal team ensured that the project satisfied all data protection privacy guidelines in Europe. The dosimetry team started planning on Friday 21^st^ May and worked through the weekend. All Category 1 patients were rescanned, had target volumes/OARs recontoured and replanned and most were back on treatment on 24th May. Radiotherapy was re-established in CUH on the 30^th^ of May. There were a low number of errors, with either over- or under-estimation of the number of fractions received, relative to that recorded on the OIS, and these were corrected in all cases prior to completion of radiotherapy. A summary of individual centre information regarding the number and type of linacs, TPS and OIS information is included in the table below.

| Radiation Oncology Department | | No of Linacs | Manufacturer | Software | |
| --- | --- | --- | --- | --- | --- |
|  |  |  |  | **TPS** | **OIS** |
| SLRON | SLH | 6 | Elekta (4) Varian (2) | OMP & Monaco | ARIA and Mosaiq |
|  | BH | 4 | Varian | Eclipse | ARIA |
|  | SJH | 4 | Varian | Eclipse | ARIA |
| CUH |  | 5 | Elekta | Monaco | Mosaiq |
| GUH |  | 3 | Siemens | Monaco | Mosaiq |
| TPS: Treatment Planning System, OIS: Oncology Information System, SLRON: St Lukes Radiation Oncology Network, SLH: St Luke's Hospital, BH: Beaumont Hospital, SJH: St James's Hospital, CUH: Cork University Hospital, GUH: Galway University Hospital | | | | | |
|  |  |  |  |  |  |

Completed NCCP Risk Assessment Templates

**NCCP Risk Assessment Tool for Radiation Oncology during the HSE IT Cyber Attack**

| Service | Challenges | Risk of the contingency | Risk of break in treatment | Consequence of decision | Subsequent risk |
| --- | --- | --- | --- | --- | --- |
| Cork University Hospital(CUH)  Age 51: Diffuse Large B cell (non GCB subtype) lymphoma of left orbit and maxillary antrum  Patient had chemotherapy with CNS prophylaxis and had completed 4/15 fractions of planned radiation at the time of the cyberattack | No access to clinical information  Main challenge was no access to the patient’s PET/CT images to replan treatment until 27/5/2021 | Contingency: Transferred patient to Bon Secours/ UPMC  Planning not finalised until PET/CT available which introduced a further delay to restarting treatment  Risk of dose fractionation error as relying on patient and doctor’s recollection of fractions delivered | Category 2 patient, but aggressive high grade lymphoma and radiation essential component of treatment so concern re loss of local control and cure for this patient | Patient restarted radiotherapy 28/5/2021 which was earlier than if I had waited for treatment to resume in CUH  Was treated 6 days a week until radiotherapy completed. No compensation required for gap | Risk of reduction in local control and consequently cure for this patient who had a 10 day gap during radiotherapy |

Fig 2a Patient with lymphoma

**NCCP Risk Assessment Tool for Radiation Oncology during the HSE IT Cyber Attack**

| Service | Challenges | Risk of the contingency | Risk of break in treatment | Consequence of decision | Subsequent risk |
| --- | --- | --- | --- | --- | --- |
| Cork University Hospital(CUH)  76 year old man with metastatic squamous cell carcinoma (unknown primary)with metastatic bone disease). Fracture of T3  Not a candidate for surgical management | Presented on 28/5/2021. Severe pain in upper thoracic spine and left shoulder. Clinical concern: impending spinal cord compression given radicular nature of pain. Dexamethasone commenced  Previous radiotherapy to mediastinum 30Gy/15+6Gy boost following chemotherapy in 2016 for a high grade lymphoma  No access to previous plan or clinical notes  No access to imaging that had demonstrated early cord compression | Contingency: Transferred patient to Bon Secours/UPMC for radiotherapy  From recollection, the cord in vicinity of T3 would have received a significant dose in 2016  Risk of overdose of spinal cord with retreatment as no information on previous max dose to the spinal cord | Break not an issue here but delaying treatment by awaiting previous radiotherapy planning information a risk | My overall impression is the risk of not treating this patient with the development of irreversible paralysis far outweighs the risk of radiation induced myelopathy  Treated patient with 30Gy/10 fractions in UPMC  Treatment commenced 2/6/2021 | Risk of myelopathy |

Fig 2b Patient treated with palliative intent

**NCCP Risk Assessment Tool for Radiation Oncology during the HSE IT Cyber Attack**

| Service | Challenges | Risk of the contingency | Risk of break in treatment | Consequence of decision | Subsequent risk |
| --- | --- | --- | --- | --- | --- |
| Cork University Hospital (CUH)  Age 52:  FIGO Stage IIIC adenocarcinoma of the cervix  Patient had 4 fractions of radiotherapy before cyber-attack. Plan was 50.4Gy/28 which included simultaneously integrated boost (SIB) to grossly enlarged node to give a dose of 55Gy/28 | No access to patient's clinical details apart from handwritten notes from Tumour Board meeting  No access to any radiology to assist with contouring for replanning | Contingency: Transfer of patient to Bon Secours /UPMC for resumption of external beam radiotherapy  Making CTV/PTV too small or too large because of lack of information and imaging  Making an error with contouring/planning due to fatigue as all staff working long hours  Making a dose fractionation error as relying on patient and Radiation Oncologist's recollection of fractions delivered | Robust evidence that prolonging overall treatment time in cervical cancer radical treatment is associated with inferior outcomes for patients | Patient resumed treatment after a gap of 6 days. (24/5/2021)  Changed to sequential rather than SIB to grossly involved node as patient’s MRI and PET/CT could be available to facilitate more accurate target volume delineation at a later stage  No compensation for gap. Patient treated 6 times per week until external beam radiotherapy completed. Brachytherapy performed on time | Risk of reduction in local control with gap in treatment  Risk of reduction in local control related to contouring and/ or planning error at time of replanning  Risk of increased rate of complications from contouring and/or planning error at time of replanning  Risk of reduction in local control with prolonging overall treatment time with SIB |

Fig 2c Patient with cervical cancer

**NCCP Risk Assessment Tool for Radiation Oncology during the HSE IT Cyber Attack**

| Service | Challenges | Risk of the contingency | Risk of break in treatment | Consequence of decision | Subsequent risk |
| --- | --- | --- | --- | --- | --- |
| Cork University Hospital(CUH)  Age 68: T2N1a grade 2 ER/PR positive, Her 2 negative invasive ductal carcinoma of left breast. Mastectomy 2/25 axillary nodes macrometastases with extra-capsular spread  Due to start radiotherapy in CUH 17/5/2021 | Not all clinical information available on this patient until 25/5/2021 | Contingency: Transferred patient to Bon Secours/UPMC for planning scan on 24/5/21  Risk of making error with contouring and/or planning as a consequence of lack of information | No break in treatment but my concern was long delay to commencing radiotherapy in a node positive breast cancer patient | Planning CT scan booked for 24/5/21 and information on histology etc became available subsequently on 25/5/21  Patient started treatment on 27/5/2021. This was before she would have started in CUH | No additional risk in transferring patient to UPMC as I had access to all information required to plan treatment commenced on the 27/5 |

Fig 2d Patient with node-positive breast cancer
